# 3-O-ethyl-L-ascorbic acid modulates pro-inflammatory cytokine and extracellular matrix-related biomarker levels in human lung fibroblasts: an *in vitro* study

**DOI:** 10.3389/abp.2026.16774

**Published:** 2026-10-01

**Authors:** Ali Türkan, Murat Türkoğlu, Gülistan Öncü, Hakan Sevinç

**Affiliations:** Biota Laboratories R&D Center, Istanbul, Türkiye

**Keywords:** 3-O-ethyl-L-ascorbic acid, collagens, extracellular matrix proteins, matrix metalloproteinases, MRC-5 cells

## Abstract

L-Ascorbic acid (also known as vitamin C) is widely used in dietary supplements and cosmetic formulations because of its antioxidant properties and critical role in numerous physiological processes. In this study, we investigated the effects of 3-O-ethyl-L-ascorbic acid (EAA), a stable vitamin C derivative, on inflammatory and extracellular matrix (ECM)-related biomarkers in MRC-5 human lung fibroblasts. We quantified protein levels of pro-inflammatory cytokines, ECM-associated proteins, and matrix-degrading enzymes using enzyme-linked immunosorbent assay (ELISA)-based assays. EAA treatment significantly reduced the basal levels of interleukin-1 Beta (IL-1β), interleukin-6 (IL-6), and tumor necrosis factor-alpha (TNF-α), indicating modulation of inflammatory signaling under unstimulated conditions. EAA also increased the levels of collagen type I (COL-I), collagen type III (COL-III), and hyaluronan synthase 2 (HAS2), suggesting enhanced production of ECM-associated components. In addition, EAA altered the levels of matrix-degrading enzymes in a time-dependent manner. Matrix metalloproteinase-1 (MMP-1) and matrix metalloproteinase-9 (MMP-9) exhibited a biphasic response characterized by higher levels at 24 h and lower levels at 48 h, whereas elastase levels were consistently reduced. Taken together, these findings suggest that EAA modulates inflammatory, ECM-associated, and ECM-degrading biomarker levels in MRC-5 fibroblasts under the tested *in vitro* conditions. Further studies are needed to determine whether these changes have functional implications for ECM homeostasis.

## Introduction

L-ascorbic acid (also known as vitamin C) is an essential water-soluble nutrient involved in numerous physiological processes in humans. Its biochemical functions primarily arise from its role as a cofactor for approximately 15 enzymes and its antioxidant properties. Specifically, vitamin C serves as a cofactor for hydroxylases, monooxygenases, and dioxygenases, which are necessary for the synthesis of collagen, carnitine, norepinephrine, and amidated peptide hormones (Nishikimi and Yagi, 1991; Bruno et al., 2006; Hacısevki, 2009; Grădinaru and Popa, 2025). As a key cellular antioxidant, L-ascorbic acid efficiently neutralizes reactive oxygen species (ROS), thereby protecting cellular components, including DNA, lipids, and proteins, from oxidative damage (Montecinos et al., 2007; Pehlivan, 2017; Gęgotek and Skrzydlewska, 2022).

The extracellular matrix (ECM) provides essential structural and biochemical support to surrounding cells and plays a critical role in maintaining tissue integrity. Vitamin C is indispensable for ECM homeostasis because vitamin C-dependent hydroxylases catalyze the hydroxylation of proline and lysine residues, a process required for the stability of the collagen triple helix. Major ECM components include collagen type I (COL-I) and collagen type III (COL-III), which provide structural support and tensile strength to connective tissues (Ricard-Blum, 2011), and hyaluronic acid (HA), a glycosaminoglycan that contributes to tissue hydration, viscoelasticity, and cell signaling. HA is primarily synthesized by hyaluronan synthase 2 (HAS2) (Jiang et al., 2007). Age-related or pathological dysregulation of ECM homeostasis is directly or indirectly implicated in numerous chronic diseases, underscoring the therapeutic potential of agents capable of promoting controlled ECM remodeling (Theocharis et al., 2016; Bonnans et al., 2014).

In the lung, the ECM is a key regulator of development, homeostasis, and tissue repair and plays an active role in modulating cellular behavior under both physiological and pathological conditions (Bonnans et al., 2014; White, 2015; Zhou et al., 2018). Pulmonary ECM is organized into two key compartments: the basement membrane and the interstitial matrix. Within the lung interstitium, resident fibroblasts are the principal ECM-producing cells and are critical for maintaining tissue architecture, coordinating repair processes, and driving ECM remodeling following injury. Through the synthesis and deposition of structural matrix proteins, particularly collagens, fibroblasts contribute to the preservation of normal lung function and the regulation of wound healing responses.

Inflammatory signaling plays an important role in regulating ECM homeostasis. Pro-inflammatory cytokines, such as interleukin-1 Beta (IL-1β), interleukin-6 (IL-6), and tumor necrosis factor-alpha (TNF-α), stimulate the production of matrix-degrading enzymes and contribute to ECM degradation and tissue remodeling (Schönbeck et al., 1998; Schwingshackl et al., 1999; Atkinson and Senior, 2003). Persistent elevation of these mediators has been implicated in chronic inflammatory and fibrotic lung diseases, highlighting the importance of factors capable of modulating both inflammatory and ECM-related pathways.

Matrix metalloproteinases (MMPs) are zinc-dependent endopeptidases that regulate physiological ECM turnover and tissue remodeling (Page-McCaw et al., 2007). MMPs degrade major ECM components, including collagen, proteoglycans, laminin, and fibronectin, which are integral constituents of the pulmonary parenchyma. In addition, elastase degrades elastin, another essential ECM component. Although elastase is classically associated with neutrophils and other inflammatory cells, elastase activity and expression have also been reported in fibroblasts, including human dermal fibroblasts, in which it has been implicated in skin aging and wrinkle formation (Homsy et al., 1988; Tsuji et al., 2001; Chua and Laurent, 2006; Gregory et al., 2015). Consequently, dysregulated protease activity, including that of MMPs and elastase, may contribute to excessive ECM degradation and has been implicated in the pathogenesis of various pulmonary disorders (Winklhofer-Roob et al., 1997; Ohbayashi, 2002; Pardo et al., 2016; Burgstaller et al., 2017; Ito et al., 2019). As a result, considerable attention has been directed toward bioactive compounds that may help preserve ECM homeostasis and mitigate tissue damage in lung diseases (Kilic et al., 2024). Consistent with these observations, vitamin C has been reported to exert protective effects in several respiratory diseases, including chronic obstructive pulmonary disease (COPD), asthma, pulmonary fibrosis, and lung cancer (Ghalibaf et al., 2023).

Vitamin C is widely used in cosmetic products and dietary supplements because of its antioxidant properties and its important roles in skin health, collagen synthesis, and overall physiological function (Li and Schellhorn, 2007; Pullar et al., 2017; Carr and Maggini, 2017). However, the chemical instability of L-ascorbic acid in aqueous environments and its relatively low cellular uptake have driven the development of more stable derivatives, such as 3-O-ethyl-L-ascorbic acid (EAA) (Iliopoulos et al., 2019). EAA exhibits enhanced chemical stability and has been reported to have lower cytotoxicity than ascorbic acid in L929 dermal fibroblasts (Golonka et al., 2017); however, its effects on human lung fibroblasts have not yet been investigated. Therefore, the present study aimed to investigate the effects of EAA on biomarkers associated with ECM synthesis (COL-I, COL-III, and HAS2), ECM degradation (MMP-1, MMP-9, and elastase), and inflammation (IL-1β, IL-6, and TNF-α) in MRC-5 human lung fibroblasts under *in vitro* conditions.

## Materials and methods

### Preparation of EAA

A stock solution of EAA (500 mM, 99.9%; Soho Aneco, Nanjing, China) was prepared by dissolving the powder in phosphate-buffered saline (PBS), followed by 5 min of vortexing to ensure complete dissolution. The prepared stock solution was aliquoted and stored at −20 °C in the dark until use.

### Cell culture

The human lung fibroblast cell line MRC-5 was obtained from the American Type Culture Collection (ATCC, Manassas, VA, USA) and cultured in Dulbecco’s Modified Eagle Medium (DMEM; Gibco, Thermo Fisher Scientific, Waltham, MA, USA) supplemented with 10% fetal bovine serum (FBS; Sigma-Aldrich, St. Louis, MO, USA), 2 mM L-glutamine (Gibco, Thermo Fisher Scientific, Waltham, MA, USA), and 1% penicillin-streptomycin (Sigma-Aldrich, St. Louis, MO, USA). The cells were maintained at 37 °C in a humidified atmosphere containing 5% CO_2_. The culture medium was replaced every 2–3 days, and cells were passaged at 80%–90% confluency.

### Cell viability assay

Cell viability and the determination of non-toxic EAA concentrations were assessed using the 2,3-bis(2-methoxy-4-nitro-5-sulfophenyl)-2H-tetrazolium-5-carboxanilide (XTT) assay (Roche Diagnostics, Mannheim, Germany), following the manufacturer’s instructions. MRC-5 cells were seeded in 96-well plates at a density of 10^4^ cells/well and incubated overnight for attachment. The cells were then treated with EAA at concentrations ranging from 1.25 to 160 mM for 24 and 48 h. After treatment, 50 µL of the XTT labeling mixture was added to each well and incubated for 4 h at 37 °C. Absorbance was measured at 490 nm with a reference wavelength of 650 nm using a microplate reader (Bio-Rad Laboratories, Hercules, CA, USA). Cell viability was expressed as a percentage relative to the untreated control group.

### EAA treatment for protein analysis

MRC-5 cells were seeded at a density of 10^5^ cells/well in 6-well microplates and incubated overnight to allow cell attachment. To determine the protein levels of IL-6, IL-1β, and TNF-α, the cells were treated with 5 mM EAA for 24 h. For the measurement of MMP-1, MMP-9, elastase, HAS2, COL-I, and COL-III, the cells were treated with 5 mM EAA for 24 and 48 h. After the incubation period, the cell culture supernatants were collected and centrifuged to remove cell debris. The clarified supernatants were aliquoted and stored at −80 °C until further analysis.

### ELISA analysis

Cell numbers were counted for normalization across samples. The levels of IL-1β (Cat. No. YLA1539HU), IL-6 (Cat. No. YLA0828HU), TNF-α (Cat. No. YLA1337HU), MMP-1 (Cat. No. YLA0330HU), MMP-9 (Cat. No. YLA1678HU), HAS2 (Cat. No. YLA0649HU), elastase (Cat. No. YLA1650HU), COL-I (Cat. No. YLA1182HU), and COL-III (Cat. No. YLA0134HU) in cell culture supernatants were measured using commercial ELISA kits (YL Biont, Shanghai, China), according to the manufacturer’s instructions. Absorbance was recorded at 450 nm using a microplate reader (Bio-Rad, Hercules, CA, USA). All assays were performed in biological triplicates.

### Statistical analysis

Experiments were performed with three or four independent biological replicates, depending on the assay; data are presented as the mean ± standard deviation (SD). Statistical analyses were performed separately for each time point and assay condition. Differences between the control and treatment groups were analyzed using a Student's t-test or a two-way ANOVA followed by a Tukey’s *post hoc* test, as appropriate. A p-value of <0.05 was considered statistically significant, and exact *p-values* are reported in the text and figure legends where applicable. All statistical analyses were performed using OriginLab software (version 10.25).

## Results

### Effect of EAA treatment on cell viability

Cell viability was assessed following exposure to increasing concentrations of EAA (1.25–160 mM) for 24 h and 48 h (Figure 1). At low concentrations (1.25–5 mM), viability at 24 h slightly exceeded control values (110%–120%), suggesting a potential stimulatory effect on cell proliferation. However, prolonged exposure (48 h) reduced viability to near control levels. At intermediate concentrations (10–20 mM), viability progressively declined, with reductions of approximately 10%–25% compared to the control group. High concentrations (≥40 mM) caused marked cytotoxicity. Based on these findings, 5 mM was selected for subsequent experiments, as this concentration maintained cell viability at levels comparable to or slightly above the control and did not induce cytotoxic effects following either 24 or 48 h of exposure. In contrast, higher concentrations (≥10 mM) were associated with a progressive decline in cell viability.

**FIGURE 1:**
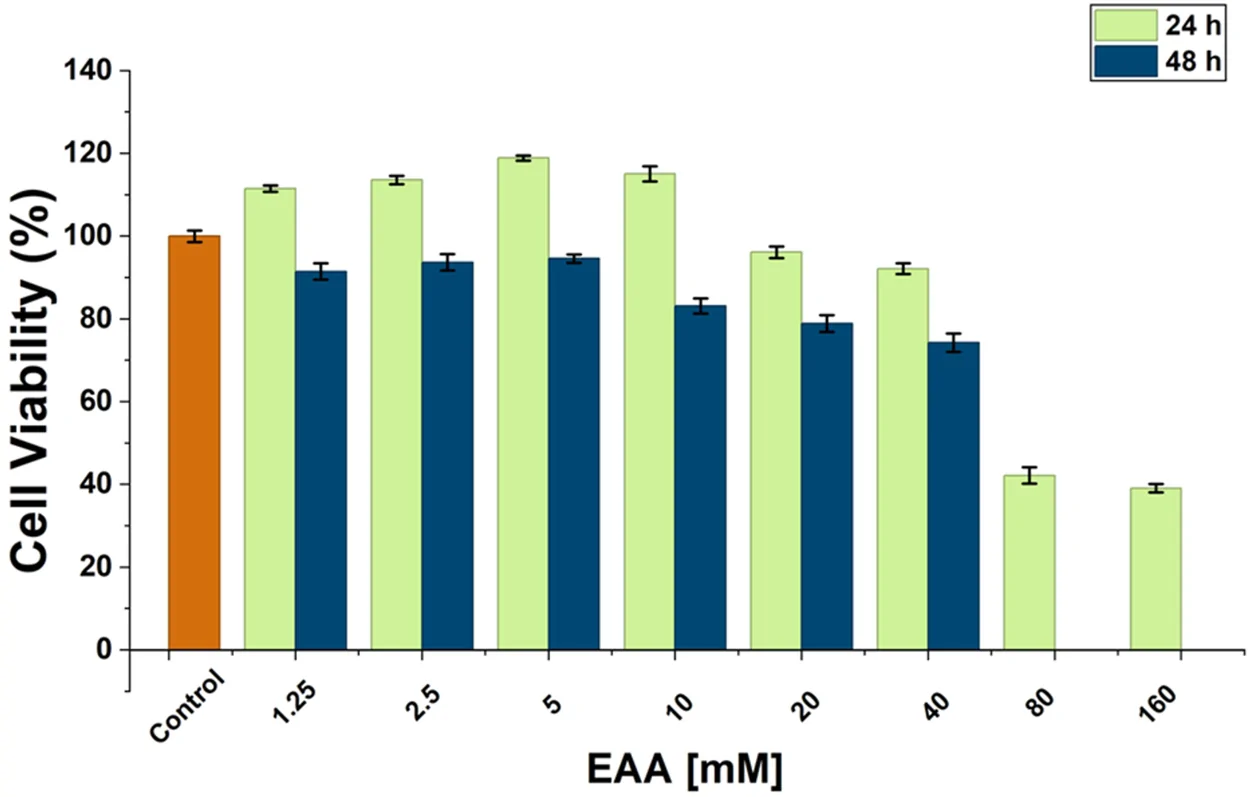
Effect of EAA on MRC-5 fibroblast cell viability. Cells were treated with increasing concentrations of EAA (1.25–160 mM) for 24 h (light green) and 48 h (dark blue). Control cells (orange) were maintained without treatment. Cell viability was assessed using the XTT assay and expressed as a percentage relative to untreated controls. Data represent the mean ± SD of three independent experiments.

### Effect of EAA treatment on basal levels of pro-inflammatory cytokines in MRC-5 cells

The effect of EAA on basal pro-inflammatory cytokine production was evaluated by measuring IL-1β, IL-6, and TNF-α levels in MRC-5 fibroblasts. EAA treatment resulted in a small but statistically significant decrease in IL-1β levels, with a reduction of approximately 3–4% compared with the control group (*p* = 0.024) (Figure 2A). In contrast, IL-6 levels were markedly reduced following EAA exposure, showing an approximate 26% decrease relative to the control group (*p* = 0.013) (Figure 2B). TNF-α levels were also significantly lower in EAA-treated cells than in untreated controls, with an approximate 24% reduction (*p* = 0.006) (Figure 2C). Collectively, these findings suggest that EAA decreases basal pro-inflammatory cytokine production in MRC-5 fibroblasts, with more pronounced effects on IL-6 and TNF-α than on IL-1β under the tested *in vitro* conditions.

**FIGURE 2:**
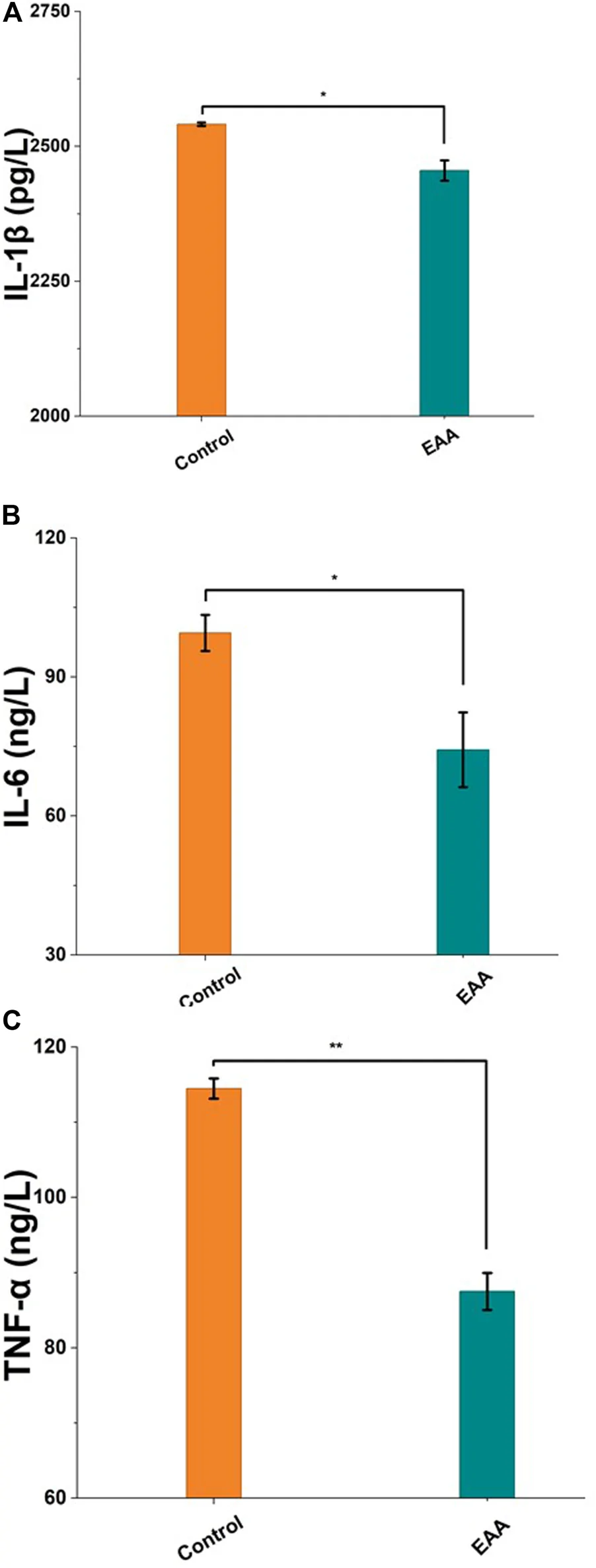
Effect of EAA Treatment on Basal Pro-Inflammatory Cytokine Levels in MRC-5 Fibroblasts. Panels show IL-1β **(A)**, IL-6 **(B)**, and TNF-α **(C)** levels in the absence (control) and presence of 5 mM EAA. Experiments were performed as described in the Materials and Methods section. Data are presented as mean ± SD (*n* = 3). Statistical analyses were performed using Student’s t-test, and significance levels are indicated above the bars (*p < 0.05, **p < 0.01, ***p < 0.001).

### Effect of EAA treatment on the levels of ECM-related markers COL-I, COL-III, and HAS2 in MRC-5 cells

To evaluate the effect of EAA on ECM-associated markers, COL-I and COL-III protein levels were measured in MRC-5 cells. As shown in Figures 3A,B, EAA treatment significantly enhanced the levels of COL-I and COL-III compared with the untreated control group, and these effects were influenced by incubation time. For COL-I (Figure 3A), EAA significantly increased COL-I levels at both 24 and 48 h. The stimulatory effect was more pronounced after 48 h, with EAA-treated cells exhibiting the highest COL-I levels. Two-way ANOVA revealed significant main effects of treatment (*p* < 0.0001) and time (*p* < 0.0001), and a significant treatment × time interaction (*p* < 0.0001). Tukey’s *post hoc* analysis confirmed significantly higher COL-I levels in EAA-treated cultures than in the corresponding controls at 24 h (*p* = 0.032) and 48 h (*p* < 0.0001), with the largest increase observed in the 48-h EAA-treated group.

**FIGURE 3:**
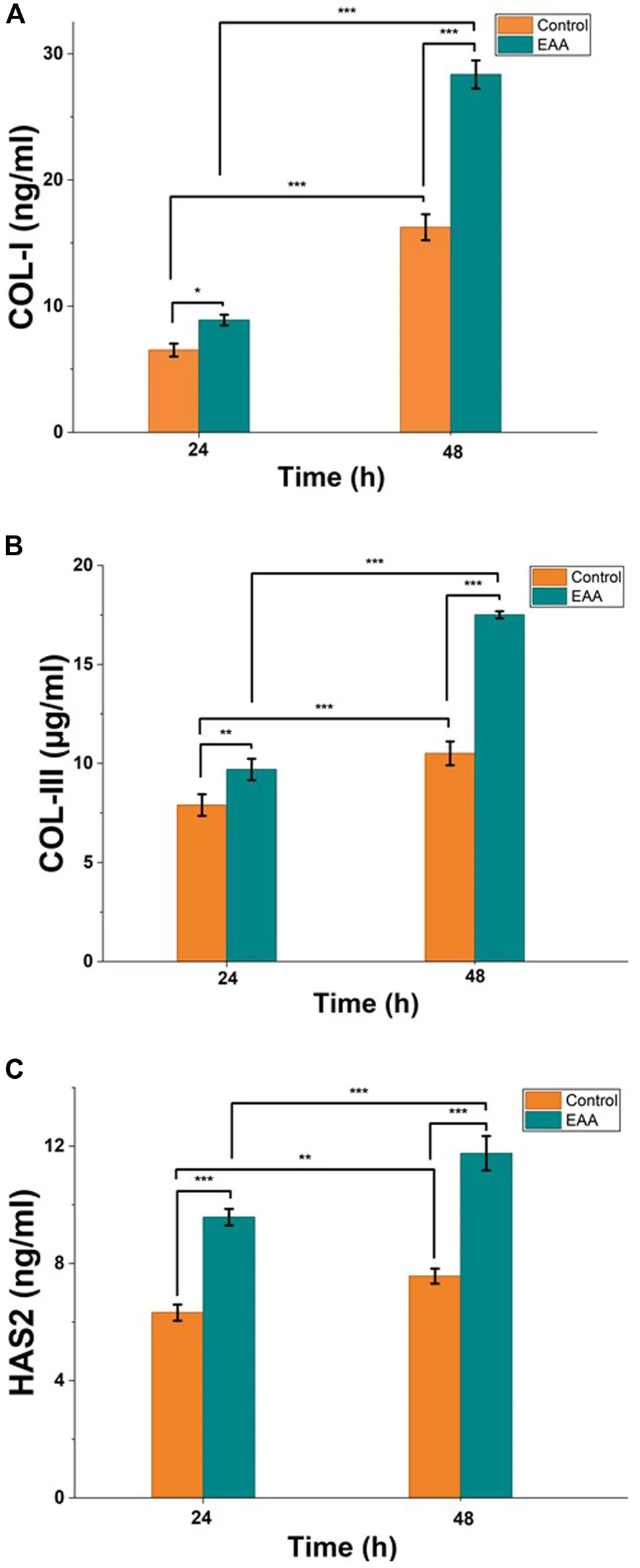
Effect of EAA Treatment on Extracellular Matrix-Associated Biomarker Levels in MRC-5 Fibroblasts. **(A)** COL-I, **(B)** COL-III, and **(C)** HAS2 levels in control and EAA (5 mM)-treated cells after 24 and 48 h of incubation. Experiments were performed as described in the Materials and Methods section. Data are presented as mean ± SD (*n* = 3). Statistical analyses were performed using two-way ANOVA followed by Tukey’s multiple-comparison test. Statistical significance is indicated as follows: *p < 0.05, **p < 0.01, ***p < 0.001.

Similarly, COL-III levels were significantly elevated by EAA treatment (Figure 3B). Two-way ANOVA demonstrated significant effects of treatment (*p* < 0.0001), time (*p* < 0.0001), and treatment × time interaction (*p* < 0.0001). Post hoc analysis showed that EAA significantly increased COL-III levels after 24 h (*p* = 0.009) and produced a markedly greater increase after 48 h (*p* < 0.0001), indicating a time-dependent enhancement of COL-III synthesis.

In addition to collagens, HAS2 levels were also evaluated following EAA treatment. As shown in Figure 3C, HAS2 levels followed a pattern similar to that observed for COL-I and COL-III. EAA treatment significantly increased HAS2 levels at both time points, with the highest levels detected after 48 h of treatment. Two-way ANOVA revealed significant effects of treatment (*p* < 0.0001) and time (*p* < 0.0001), and a significant treatment × time interaction (*p* = 0.0288). Tukey’s multiple comparison test confirmed significant differences between the EAA-treated and control groups at both 24 and 48 h (both *p* < 0.0001).

Overall, these findings demonstrate that EAA significantly increases the levels of ECM-associated markers, including COL-I, COL-III, and HAS2, with the strongest effects observed after 48 h of treatment.

### Effect of EAA on the protein levels of matrix-degrading enzymes MMP-1, MMP-9, and elastase in MRC-5 cells

The integrity of the ECM is regulated by a balance between matrix synthesis and degradation. Matrix metalloproteinases (MMPs) and elastase are key enzymes involved in ECM remodeling and degradation. MMP-1 degrades fibrillar collagens, including COL-I and COL-III, whereas MMP-9 participates in the degradation of various ECM components. Elastase contributes to the breakdown of elastic fibers, thereby affecting tissue elasticity. To further evaluate the potential effects of EAA on ECM homeostasis, we examined its influence on MMP-1, MMP-9, and elastase levels in MRC-5 cells.

As shown in Figure 4A, EAA treatment affected MMP-1 levels in a time-dependent manner. At 24 h, MMP-1 levels were higher in EAA-treated cells than in control cells. In contrast, after 48 h, EAA treatment reduced MMP-1 levels compared with the corresponding control group. A two-way ANOVA revealed a significant effect of time (*p* < 0.0001) and a significant treatment × time interaction (*p* = 0.0041), whereas the main effect of treatment was not significant (*p* = 0.538). These findings indicate that the effect of EAA on MMP-1 levels depended on the duration of treatment.

**FIGURE 4:**
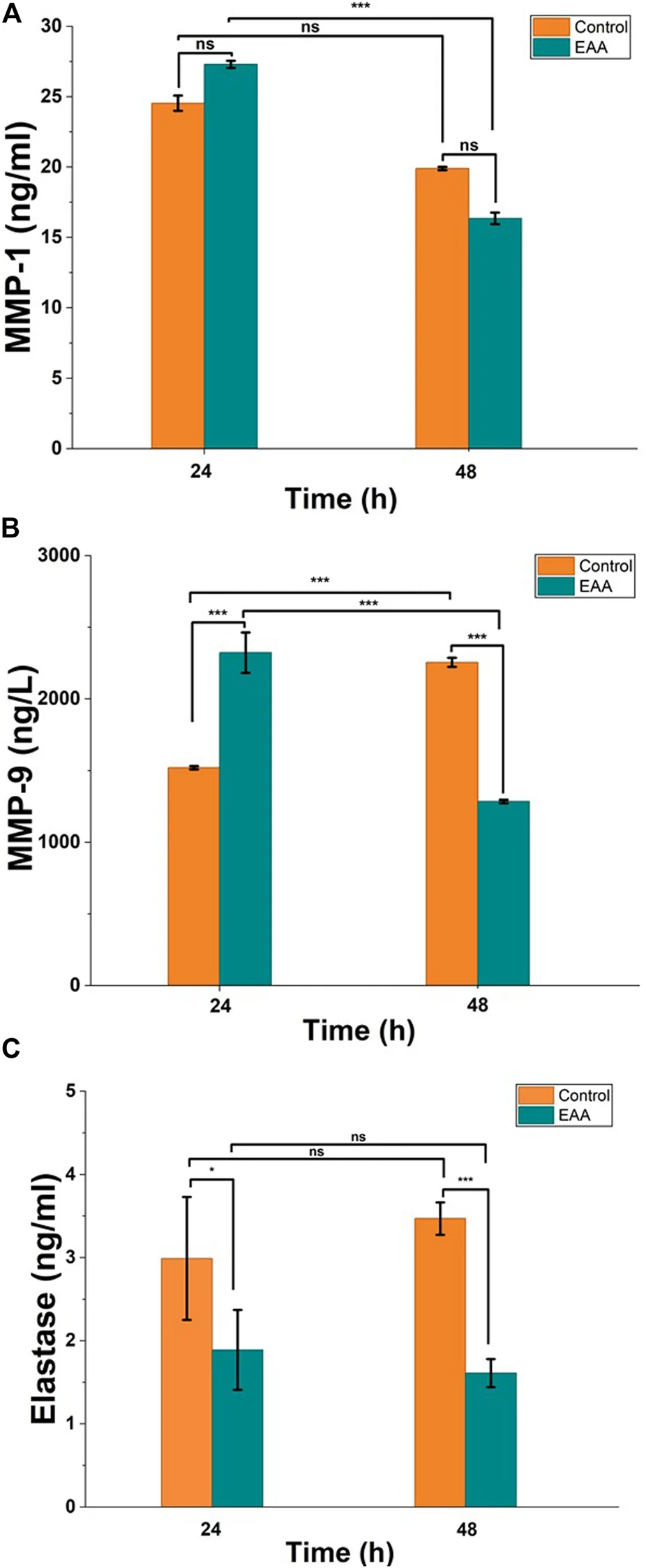
Effect of EAA Treatment on ECM Degradation-Associated Protein Levels in MRC-5 Fibroblasts. **(A)** MMP-1, **(B)** MMP-9, and **(C)** elastase levels in control and EAA (5 mM)-treated cells after 24 and 48 h of incubation. Experiments were performed as described in the Materials and Methods section. Data are presented as mean ± SD (*n* = 4). Statistical analyses were performed using two-way ANOVA followed by Tukey’s multiple-comparison test. Statistical significance is indicated as follows: *p < 0.05, **p < 0.01, ***p < 0.001, ns: not significant.

A similar time-dependent response was observed for MMP-9 (Figure 4B). At 24 h, EAA treatment significantly increased MMP-9 levels compared with the untreated control group. However, following 48 h of treatment, MMP-9 levels were markedly reduced in EAA-treated cells relative to the corresponding control group. Two-way ANOVA demonstrated significant effects of treatment (*p* = 0.0393), time (*p* = 0.0013), and treatment × time interaction (*p* < 0.0001). The highly significant interaction confirms that the response of MMP-9 to EAA differed substantially between the two incubation periods.

In contrast, EAA consistently reduced elastase levels at both time points (Figure 4C). A two-way ANOVA revealed a significant main effect of treatment (*p* < 0.0001), whereas neither the effect of time (*p* = 0.512) nor the treatment × time interaction (*p* = 0.175) was significant. Tukey’s multiple comparison test confirmed significantly lower elastase levels in EAA-treated cells than in control cells at both 24 h (*p* = 0.0247) and 48 h (*p* = 0.0008).

Overall, EAA significantly affected the levels of MMP-1, MMP-9, and elastase in MRC-5 fibroblasts. The effects on MMP-1 and MMP-9 were dependent on treatment duration, whereas elastase levels were consistently reduced following EAA treatment.

## Discussion

The present study examined the effects of EAA on cell viability and selected inflammatory and ECM-related biomarkers in human MRC-5 lung fibroblasts. At the concentration selected for the biomarker experiments (5 mM), EAA did not reduce cell viability after 24 or 48 h of exposure. These findings support the use of 5 mM EAA under the conditions tested and are broadly consistent with the favorable tolerability of EAA previously reported in L929 fibroblast cultures (Golonka et al., 2017).

EAA reduced the basal levels of IL-1β, IL-6, and TNF-α in MRC-5 cells. These cytokines are involved in inflammatory signaling and can promote ECM degradation by regulating matrix-degrading enzymes (Schönbeck et al., 1998; Atkinson and Senior, 2003; Manicone and McGuire, 2008). The observed reductions are therefore consistent with a potential effect of EAA on basal inflammatory signaling in fibroblasts. However, the experiments were performed in the absence of an inflammatory stimulus. Accordingly, the results indicate modulation of constitutive cytokine levels rather than suppression of an induced inflammatory response. Further studies using relevant inflammatory stimuli are needed to determine whether EAA can attenuate cytokine responses in a model that more closely reflects inflammatory tissue injury.

EAA also increased COL-I and COL-III protein levels in MRC-5 cells. For both collagen types, two-way ANOVAs revealed significant effects of treatment and time and significant treatment × time interactions, indicating that the response to EAA depended on exposure duration. Although collagen levels increased over time in both control and EAA-treated cultures, the increases were more pronounced in EAA-treated cells, particularly after 48 h. The observed increases in COL-I and COL-III are consistent with the established role of vitamin C in collagen biosynthesis as a cofactor for prolyl and lysyl hydroxylases, enzymes that are essential for the post-translational modification and structural stabilization of collagen molecules (Pullar et al., 2017). Furthermore, the present findings agree with those of Chan et al. (1990), who reported that ascorbic acid stimulates both type I and type III collagen synthesis in fibroblasts, with collagen production reaching maximal levels after approximately 2 days of exposure. The stronger responses observed at 48 h than at 24 h in the present study are consistent with this temporal pattern. In addition to its role in collagen maturation, vitamin C has been shown to promote collagen gene expression. Phillips et al. (1994) demonstrated that ascorbic acid enhances collagen biosynthesis in human dermal fibroblasts by increasing the expression of type I and type III collagen mRNAs, suggesting regulation at the pretranslational level.

In addition to increasing collagen levels, EAA treatment significantly increased HAS2 levels in MRC-5 fibroblasts. HAS2 levels were higher in EAA-treated cells than in controls at both time points, with a greater increase after longer exposure, supporting a time-dependent response. These findings suggest that EAA may promote hyaluronic acid-related biosynthetic processes in fibroblasts. The observed increase in HAS2 is consistent with previous reports that ascorbic acid can stimulate hyaluronic acid synthesis. For example, Schachtschabel and Binninger (1993) reported enhanced hyaluronic acid production in cultured normal and glaucomatous human trabecular meshwork cells following ascorbic acid treatment.

A notable finding of this study was the time-dependent regulation of MMP-1 and MMP-9 by EAA. A two-way ANOVA revealed significant treatment × time interactions for both MMP-1 and MMP-9, indicating that the effects of EAA on MMP levels depended on exposure duration. For MMP-1, a significant interaction was observed despite the absence of a significant overall treatment effect, suggesting that EAA influenced MMP-1 levels differently over time rather than exerting a consistent stimulatory or inhibitory effect. In contrast, MMP-9 showed significant effects of treatment and time and a significant treatment × time interaction, indicating a more pronounced response to EAA. For both MMPs, EAA-treated cells exhibited higher levels at an earlier time point and lower levels after prolonged exposure, consistent with a biphasic temporal response. The biological significance of this pattern remains to be established. MMP levels are dynamically regulated during tissue remodeling, during which temporal changes in matrix degradation often accompany subsequent alterations in matrix synthesis (Page-McCaw et al., 2007; Manicone and McGuire, 2008; Lee and Kim, 2022).

EAA treatment also significantly reduced elastase levels in MRC-5 fibroblasts. Elastase is a proteolytic enzyme that contributes to the degradation of elastin and other ECM components and has been implicated in tissue injury and progressive structural deterioration, particularly in the lung (Chua and Laurent, 2006; Döring, 1994). A two-way ANOVA revealed a significant effect of treatment, whereas neither the effect of time nor the treatment × time interaction was significant. These findings indicate that EAA consistently reduced elastase levels regardless of exposure duration. In line with this observation, elastase levels were lower in EAA-treated cells than in control cells at both 24 and 48 h. Although elastase is most commonly associated with neutrophils and other inflammatory cells (Chua and Laurent, 2006; Gregory et al., 2015), elastase expression has also been reported in human dermal fibroblasts, where it has been linked to wrinkle formation and skin aging (Homsy et al., 1988; Tsuji et al., 2001).

The concurrent increase in collagen- and HAS2-related protein levels, together with the lower MMP-1, MMP-9, and elastase levels observed at 48 h, is consistent with a shift in the measured biomarkers toward an ECM-preserving profile. This observation may be relevant because the lung ECM is not merely a structural scaffold but a dynamic regulator of tissue homeostasis, repair, and cell behavior, with tightly controlled matrix synthesis and degradation being essential for normal lung function (Zhou et al., 2018). Increased production of structural matrix proteins and glycosaminoglycan-related components, accompanied by reduced levels of ECM-degrading enzymes, may therefore reflect a cellular environment that favors matrix maintenance. However, this interpretation remains provisional because the study did not directly assess protease activity, collagen deposition or cross-linking, hyaluronan production, or functional ECM remodeling. Furthermore, because the experiments were conducted in a single fibroblast cell line under *in vitro* conditions, caution is warranted when extrapolating these findings to more complex tissue environments. This limitation is particularly relevant to lung tissue, where fibroblasts operate within a multicellular and dynamically regulated microenvironment. In addition, L-ascorbic acid was not included as a comparator control. Therefore, although EAA significantly modulated inflammatory and ECM-related biomarkers, the present study does not permit direct comparison of its effects with those of native vitamin C. Future studies should include side-by-side comparisons with L-ascorbic acid, and other stable vitamin C derivatives, such as 2-phospho-L-ascorbate, to better define the relative biological activities and potential advantages of EAA.

In conclusion, EAA modulated basal pro-inflammatory cytokine levels and ECM-related biomarkers in MRC-5 fibroblasts under the tested *in vitro* conditions. The observed reductions in pro-inflammatory cytokines, increases in COL-I, COL-III, and HAS2 levels, and the modulation of MMPs and elastase suggest that EAA may influence pathways involved in extracellular matrix homeostasis. Further studies are required to determine the functional significance of these findings and their relevance to ECM remodeling in more complex biological systems.

## Data Availability

The raw data supporting the conclusions of this article will be made available by the authors, without undue reservation.
